# Interoperability Between Mobile Money Agents and Choice of Network Operators: The Case of Tanzania

**DOI:** 10.1515/rne-2023-0024

**Published:** 2023-09-01

**Authors:** Lukasz Grzybowski, Valentin Lindlacher, Onkokame Mothobi

**Affiliations:** Faculty of Economic Sciences, University of Warsaw, 44/50 Dluga Street, 00241 Warsaw, Poland; School of Economics, University of Cape Town, Rondebosch, 7701, Cape Town, South Africa; TU Dresden, Ifo Institute Munich & CESifo, Helmholtzstr. 10, 01069 Dresden, Germany; School of Governance, University of Witwatersrand, 2 St Davids PI, Parktown, Johannesburg, South Africa; University of Botswana, Gaborone, Botswana

**Keywords:** mobile money, interoperability; M-Pesa; Tanzania; mobile networks, O16, O18, O33, L86, L96

## Abstract

In this paper, we investigate the effects of non-exclusive agreements between networks of mobile money agents on mobile network operator choices, using survey data from Tanzania conducted in 2017. By combining survey responses with geo-location data and information on agent proximity, we employ discrete choice models to analyze consumers’ decisions in subscribing to mobile network operators and their corresponding mobile money providers. Our findings highlight the significant influence of the distance to mobile money agents on consumers’ subscription choices. To explore the impact of interoperability (non-exclusivity) at the mobile money agent level, where consumers can use the nearest agent from any mobile money provider, we assess its effects on market shares of mobile network operators. Our results indicate that interoperability at the agent level has only a minor impact on market shares. Smaller operators experience marginal gains as their consumers can now utilize agents of larger providers, which are often closer in proximity. In conclusion, we find that interoperability at the agent level does not considerably alter the market structure in the context Tanzania during the period under consideration.

## 1 Introduction

Mobile telecommunications present a significant opportunity to foster economic growth in developing countries, particularly by bridging the gap between the affluent and the underprivileged through the provision of essential services to remote areas. Mobile phones facilitate access to services such as health information, education programs, financial services, and more, which are typically inaccessible to people in remote regions. One remarkable service in this regard is the mobile money platform, widely recognized as M-Pesa.1M-Pesa, a mobile money service that relies on mobile network infrastructure, experienced rapid growth in numerous countries in Africa and beyond, following its launch in Kenya in 2007 under the name of M-Pesa (see Mothobi and Grzybowski 2017).


The mobile money service enables users to conduct transactions through a mobile account linked to their unique mobile phone number. It empowers individuals to deposit, withdraw, transfer money locally and internationally, and also access credit, savings, and insurance services. It is essential to distinguish mobile money from mobile banking, where users depend on internet-enabled mobile devices to manage their traditional bank accounts. In Sub-Saharan Africa, mobile money services are primarily offered by mobile network operators, which compete with traditional banking establishments.2On the contrary, in China, mobile financial services are predominantly provided by third-party payment service providers like Alipay and WeChat, offering smartphone apps linked to bank or financial institution accounts.


A survey conducted in Tanzania by Research ICT Africa in 2017, which we use in our analysis, revealed that 55 % of respondents utilized mobile money, while only 17 % had a bank account. This disparity in mobile money usage compared to traditional bank accounts is even more pronounced in other countries across Sub-Saharan Africa. According to the 2021 Global Findex report from the World Bank, 33 % of adults in Sub-Saharan Africa held a mobile money account, in contrast to 10 % of adults globally. Furthermore, all 11 economies worldwide, where more adults possess only a mobile money account rather than a financial institution account, are located in Sub-Saharan Africa.3These countries include Benin, Cameroon, the Republic of Congo, Cote d’Ivoire, Gabon, Guinea, Malawi, Sierra Leone, Tanzania, Zambia, and Zimbabwe. These figures underscore the critical role mobile money plays in facilitating financial inclusion across Sub-Saharan Africa.

Despite the widespread use of mobile money in Sub-Saharan Africa, digital payments have not yet become commonplace for everyday purchases at local stores and marketplaces. Consequently, mobile money users often have to rely on cash-in and cash-out points, such as bank agents, mobile money agents, or automated teller machines (ATMs), to deposit and withdraw cash. To attract more users, mobile money operators must invest in building a robust network of agents across the country. The availability and proximity of these agents play a crucial role in the choice of a mobile money provider, with early movers and large network operators enjoying a competitive advantage over smaller players.

An illustrative example of the challenges faced in this context comes from Kenya in 2012, where Airtel, the second-largest mobile network operator, lodged a complaint with the Competition Authority of Kenya (CAK) against Safaricom, the market leader. The complaint sought to remove the exclusive arrangements Safaricom had with agents and grant access to its agent network to rival mobile network operators. Airtel also argued that Safaricom was leveraging its dominant position by charging higher fees for mobile cash transfers to Airtel customers compared to transactions between Safaricom accounts. Safaricom’s defense rested on the substantial investments made in developing its agent network, claiming that opening it up to competitors would be unfair. Eventually, in 2014, the CAK ordered Safaricom to open its M-Pesa agent network to other mobile money providers.4Similarly, MTN Uganda, one of the largest mobile network operators in the country, faced penalties in 2016 for refusing to provide services to another mobile money operator, EzeeMoney. This move towards interoperability aimed at mitigating market domination.

Interoperability can be achieved at various levels. Firstly, there is account-to-account (A2A) interoperability, which enables mobile money customers to transfer funds between accounts held at different mobile money providers or between a mobile money provider and a bank. In such case agents have non-exclusive agreements with mobile money providers. Secondly, interoperability at the agent level allows agents to represent multiple mobile money providers. Thirdly, interoperability at the merchant level enables consumers to transact at any retailer, regardless of the account held by the merchant. Lastly, interoperability at the mobile network level enables subscribers of one network operator to access mobile money services provided by another network operator.

This paper focuses on examining the impact of interoperability at the level of agents, specifically the effects of non-exclusivity agreements between agents and mobile money providers, on the market shares of mobile operators. To conduct this analysis, we utilize extensive survey data collected from 1200 individuals in Tanzania by Research ICT Africa in 2017. By incorporating geo-location data of respondents, we merge the survey information with details about the proximity of mobile money agents and mobile network infrastructure obtained from *finmark*. Additionally, we calculate the distance from each household to mobile money agents and mobile network towers.

To estimate the effects, we employ discrete choice models, wherein consumers’ decisions to subscribe to a particular mobile network operator (and mobile money provider) are influenced by the distance to the mobile money agent of that provider. We also include nighttime light intensity data as a proxy for economic development variations across locations and incorporate a rich set of individual characteristics.

The results indicate that the distance to the mobile money agent significantly influences subscription decisions. We use these estimates to perform counterfactual simulations, examining the consequences of implementing interoperability at the level of mobile money agents. In this hypothetical scenario, consumers would have the option to use the nearest agent from any mobile money provider. Our simulations reveal that such interoperability has only a minor impact on the market shares of mobile network operators, with smaller operators gaining slightly due to their consumers’ increased access to agents of larger providers, which are usually closer in proximity. However, it is essential to note that these findings are specific to Tanzania, where interoperability at the account-to-account level had been in place since 2016, and the Bank of Tanzania mandated the non-exclusivity of mobile money agents in 2015. To the best of our knowledge, this study is the first to empirically address the role of interoperability between mobile money providers.

The rest of the paper is structured as follows: In Section 2, we review the pertinent literature; Section 3 discusses the evolution of mobile money services in Tanzania and the broader context. Next, in Section 4, we introduce the data sets utilized in our analysis. Subsequently, Section 5 presents the econometric model we adopt, and Section 6 presents the estimation results. Finally, we conclude in Section 7.

## 2 Literature Review

Our paper contributes to various streams of literature. Firstly, it adds to the well-established theoretical literature on compatibility (interoperability) between networks. Consensus in this literature suggests that in network effect markets, welfare is maximized when all providers interconnect (see Carter and Wright 1999; Economides and Woroch 1992; Laffont et al. 1996). However, dominant firms may strategically decide not to interconnect with other networks, limiting access and competition, to preserve their market position and prevent potential competitors from gaining access to their customer base (see Evans and Schmalensee 1995).

Besen and Farrell (1994) conclude that firms tend to choose similar compatibility strategies when they are symmetric, but conflicting strategies emerge when asymmetry exists. In particular, late entrants seek to join the network of an industry leader, while the leader attempts to prevent such interconnection. Similarly, Chen et al. (2009) find that firms in symmetric markets tend to choose compatibility to expand the market, while dominant firms may opt for incompatibility in asymmetric markets.

The mentioned studies have not fully explored the tradeoff between compatibility benefits and the advantages of variety. Addressing this gap, Farrell and Saloner (1986) examine the tradeoff and highlight the possibility of excessive standardization leading to inefficiencies, particularly when unique equilibria are not focal points. They argue that if an inefficient equilibrium is reached, increasing variety and consumer welfare can be achieved with less standardization. In contrast, Farrell and Saloner (1986) present a different perspective, demonstrating that product compatibility enhances product variety for consumers, thereby increasing consumer welfare by enabling them to mix and match products. They also find that firms have incentives to produce compatible products even in non-network effect markets. When firms produce compatible products, a decrease in one firm’s price can lead to increased sales for other firms producing compatible products, creating a positive feedback loop that benefits consumers.

Moreover, Doganoglu and Wright (2006) contribute to this discussion by developing a theoretical model that examines the effect of product compatibility and multi-homing on competition. Their findings suggest that consumers enjoy greater network benefits when competing firms make their products compatible. Interestingly, they also find that similar benefits arise when firms produce incompatible products, provided consumers engage in multi-homing behavior. However, they further conclude that multi-homing may reduce competition compared to product compatibility. Collectively, these studies shed light on the complex interplay between compatibility, variety, and consumer welfare.

Few empirical studies focus on interoperability in markets such as operating systems and video games. For instance, Economides and Katsamakas (2006) study the market of PC operating systems and conclude that without interoperability, there might be a winner-takes-all outcome. In another paper, Cennamo and Santalo (2013) empirically analyze the U.S. video game industry and show that when two competing platforms pursue winner-takes-all strategies, the performance of each platform is lower.

Second, our paper contributes to the literature on mobile money. Among these studies, many focus on M-Pesa in Kenya. For instance, Mbiti and Weil (2015) analyze the use and economic impact of M-Pesa in Kenya using two waves of individual-level data on financial access. They find that M-Pesa positively impacts individual welfare by promoting banking and increasing money transfers. Jack and Suri (2014) also use two waves of about 3000 households in Kenya to study transactional networks and conclude that there is more remittance activity in households with M-Pesa users than in those without. They also find that households which use M-Pesa are more likely to remit for routine support, credit, and insurance purposes. They conclude that mobile money allows households to spread risk more efficiently through deeper financial integration and expanded informal networks. In another paper, Murendo et al. (2018) assess the effects of social network on mobile money adoption among rural households in Uganda. They find that the size of social networks positively impacts mobile money adoption. Munyegera and Matsumoto (2016) use data on 846 rural households to analyze the adoption of mobile money, remittance activity, and household welfare in Uganda. They find a positive and significant effect of mobile money access on household welfare. Similar to Jack and Suri (2014), they conclude that households that use mobile money are more likely to receive remittances than non-user households. They also find that the total value of remittances received by households that use mobile money is significantly higher than for non-user households.

The papers discussed above rely on surveys of individuals or households. There are also recent studies that apply a randomized controlled trial (RCT) to estimate the causal effects of mobile money. Randomized access to mobile money is either given directly to individuals (Batista and Vicente 2013; Batista and Vicente 2018) or small-scale entrepreneurs (Aggarwal, Brailovskaya, and Robinson 2020). In another paper, Economides and Jeziorski (2014) use mobile financial transactions among subscribers of a primary mobile phone service provider in Tanzania for three months to estimate price elasticities for different types of transactions. They find that demand for long-distance transfers is less elastic than for short-distance transfers, which suggests that mobile networks actively compete with antiquated cash transportation systems in addition to competing with each other. They use the demand estimates to provide measures of willingness to pay to avoid carrying cash in their pocket when traveling and keeping cash at home. Finally, only one qualitative paper by Bourreau and Valetti (2015) assesses the economic features of mobile payment systems in low-income countries. They conclude that mobile money can potentially drive the financial inclusion of poor households at a low cost. The theoretical literature on mobile payments and interoperability was recently reviewed by Bianchi et al. (2021).

Our contribution to the existing literature lies in our examination of interoperability at the level of mobile money agents. Specifically, we analyze the effects of compatibility, particularly non-exclusivity agreements between agents and mobile money providers, on the choices and market shares of mobile money providers in the context of Tanzania. In doing so, we investigate whether dominant mobile operators may leverage downstream agent-level interoperability in the delivery of mobile money services to reinforce their upstream position in the provision of mobile services overall.

## 3 Mobile Money and Interoperability

Mobile money and mobile banking are both financial services offered through mobile devices, but they serve different purposes and have distinct features. Mobile money services are entirely provided on mobile networks and are linked to a user’s unique mobile phone number. Users can create a mobile account, commonly known as a mobile wallet, where they can store funds. These mobile wallets enable subscribers to perform various financial services, such as domestic and international money transfers, bill payments, airtime top-ups, and more. Transactions are settled through a network of agents established by the mobile money providers, who act as intermediaries in facilitating cash-in and cash-out services.

In contrast, mobile banking necessitates users to have a bank account with the respective financial institution offering the service. It extends the capabilities of traditional banking to the mobile platform, enabling users to access their existing bank accounts through their mobile devices. Users can conduct a wide range of banking operations, including checking account balances, transferring money between accounts, paying bills, and managing investments.

As a result, mobile money gains popularity in regions with limited access to traditional banking services, providing a convenient and accessible means to manage financial transactions using basic mobile phones. On the other hand, mobile banking is more prevalent in developing countries and favored by individuals with established banking relationships, seeking the convenience of accessing their accounts on the go.

The most common mobile money service in Sub-Saharan Africa is M-Pesa, which was first launched in Kenya by Safaricom in 2007. Today, M-Pesa remains the most popular mobile money service in East African countries, including Uganda, Tanzania, Rwanda, and Burundi. Furthermore, its usage has expanded to other African countries, such as Cote d’Ivoire, Senegal, Madagascar, Mali, Niger, Botswana, Cameroon, and South Africa. Additionally, M-Pesa has found adoption outside Africa in countries like Jordan and Afghanistan. In addition to M-Pesa, several banks in Africa have introduced a similar service called e-wallet. However, there is a notable difference between e-wallet and M-Pesa. For e-wallet, the sender must have a bank account to utilize the service, while the receiver can cash out money only at ATMs using their mobile phone number and a pin.

### 3.1 Interoperability

There are different levels of interoperability. First, so-called account-to-account (A2A) interoperability enables mobile money users to transfer between two accounts held at different mobile money providers, or between a mobile money provider and a bank. Second, interoperability at the agent level refers to the ability of agents, who represent different mobile money providers, to conduct transactions on behalf of customers from various mobile money networks. In such case agents have non-exclusive agreements with mobile money providers. Third, interoperability at the merchant level allows consumers to transact at any retailer, regardless of the account held by the merchant. Lastly, interoperability at the mobile network level enables subscribers of one network operator to access mobile money services provided by another network operator.

A2A interoperability reduces transaction costs for individuals using diverse mobile money providers, leading to increased money transfer volumes and overall usage of mobile money services. Interoperability at the mobile network level empowers consumers to choose mobile money providers regardless of the mobile operator they subscribe to, which fosters increased competition in the market. At the agent level, the benefits stem from sharing the high fixed costs associated with building a network of agents, who require recruitment, training, and cash support. By serving multiple mobile money providers, these agents can achieve viability with a minimum number of customers and sufficient liquidity. Furthermore, interoperability expands the reach of agent networks, encouraging wider adoption and usage of mobile money services.

Hoernig and Bourreau (2017) suggest that interoperability is more likely to occur voluntarily when mobile money networks are still small and relatively similar in size. However, operators with a strong first-mover advantage, owing to their early start and significant investments in agent network rollout, may exhibit reluctance in opening their customer base and agent network to smaller competitors. Consequently, larger operators may face negative incentives for interoperability in the short run. Nevertheless, embracing interoperability offers substantial benefits in the medium term, particularly in markets with significant growth potential. Being a less-than-dominant operator in a large market can prove more advantageous than holding a dominant position in a small market.

A refusal to inter-operate by the largest operator can result in reduced competition, especially when the number of viable candidates for agents is limited. Smaller networks might find it economically challenging to create their own separate agent networks, further reinforcing an asymmetric market structure that negatively impacts mobile money customers. Early dominance by one operator, as exemplified by Safaricom in Kenya, may hinder other operators from achieving critical mass, even if they choose to inter-operate among themselves. In such a scenario, achieving interoperability among mobile money providers is unlikely without government intervention.

Recognizing the significance of interoperability, regulators, particularly in East African countries, have taken steps to direct mobile money operators to inter-operate. However, on a global scale, mobile money interoperability is still in its infancy stage and has yet to be introduced in other markets. Notably, in 2014, Tanzania set a pioneering example by becoming the first African country to introduce interoperability between mobile money operators. Through this initiative, operators agreed to enable mobile money senders to transfer funds directly from their wallet to the receiver’s wallet in real-time, eliminating intermediary steps and regardless of whether the transaction is on- or off-net. Likewise, in 2017, the Bank of Uganda issued a directive for all mobile money operators to ensure seamless payments across all network providers.

In 2018, Kenya achieved another significant milestone when mobile money operators, namely Safaricom, Airtel, and Telekom Kenya, reached an agreement to inter-operate. During the trial pilot stage, these operators waived any surcharges on inter-mobile money transactions, ensuring that mobile money users are charged the same amount whether they remit within or outside their networks. Inspired by the success of Tanzania’s interoperability model, the Kenyan regulator adopted a similar approach. Operators in Kenya utilize a multi-lateral agreement for the rules governing interoperability but connect bilaterally on the technical level. Building on this progress, in 2022, the Central Bank of Kenya introduced mobile money merchant interoperability.

### 3.2 Mobile Money in Tanzania

In Tanzania, there are seven mobile network operators (MNOs), where as of December 2018 the five most prominent players in terms of subscriptions were Vodacom (32 %), Tigo (29 %), Airtel (25 %), Halotel (9 %) and Zantel (3 %). These five MNOs also compete to provide mobile money services. Vodacom launched its mobile money service, M-Pesa, in April 2008, one year after the successful launch of the same service in Kenya. Also in 2008, Zantel introduced a mobile money service, Z-Pesa, which was renamed to Ezy Pesa in 2012. Airtel launched its mobile money service, Airtel Money, in 2009. Finally, Tigo introduced Tigo Pesa in September 2010. The last entrant Halotel started providing mobile money services called Halopesa in 2016.

The launch of mobile money services in Tanzania was a success and in June 2016 there were 17.3 million mobile money accounts. As of 2016, the market shares of mobile money accounts were as follows: M-Pesa (43 %), Tigo Pesa (32 %), Airtel Money (23 %), and Ezy Pesa (2 %). Mobile money platforms offer domestic and international money transfers, mobile payments (e.g. airtime top-ups), and mobile banking services (balance inquiries, withdrawals, deposits and credit services). The number of mobile money agents has also increased dramatically over time. According to the Bank of Tanzania (BoT), there were 2757 agents in Tanzania in 2008. The number of agents increased to 29,095 in 2010 and only two years later in 2012 reached 97,613. The number of agents further doubled to 203,752 in June 2014 and reached 280,675 in June 2016.

The payment services are regulated by the Bank of Tanzania, which in 2007 issued guidelines for electronic payment schemes. Based on these guidelines, banks and MNOs can offer mobile money services after receiving clearance from the BoT through a no-objection letter. Interoperability at the account, platform, and agent level was a stated requirement in the draft regulations. However, it was not formally mandated and the BoT opted for a market-based solution. Consequently, in September 2014, Airtel and Tigo reached a bilateral interoperability agreement and their off-net transfer services were launched commercially in February 2015. In December 2014, Tigo and Zantel also signed an interoperability agreement. Finally, one year later in February 2016, the market leader, Vodacom, signed bilateral agreements with Airtel and Tigo. Consequently, in 2016 Tanzania became the first country to achieve full interoperability between all its mobile money providers.

## 4 Data

**Figure 1:**
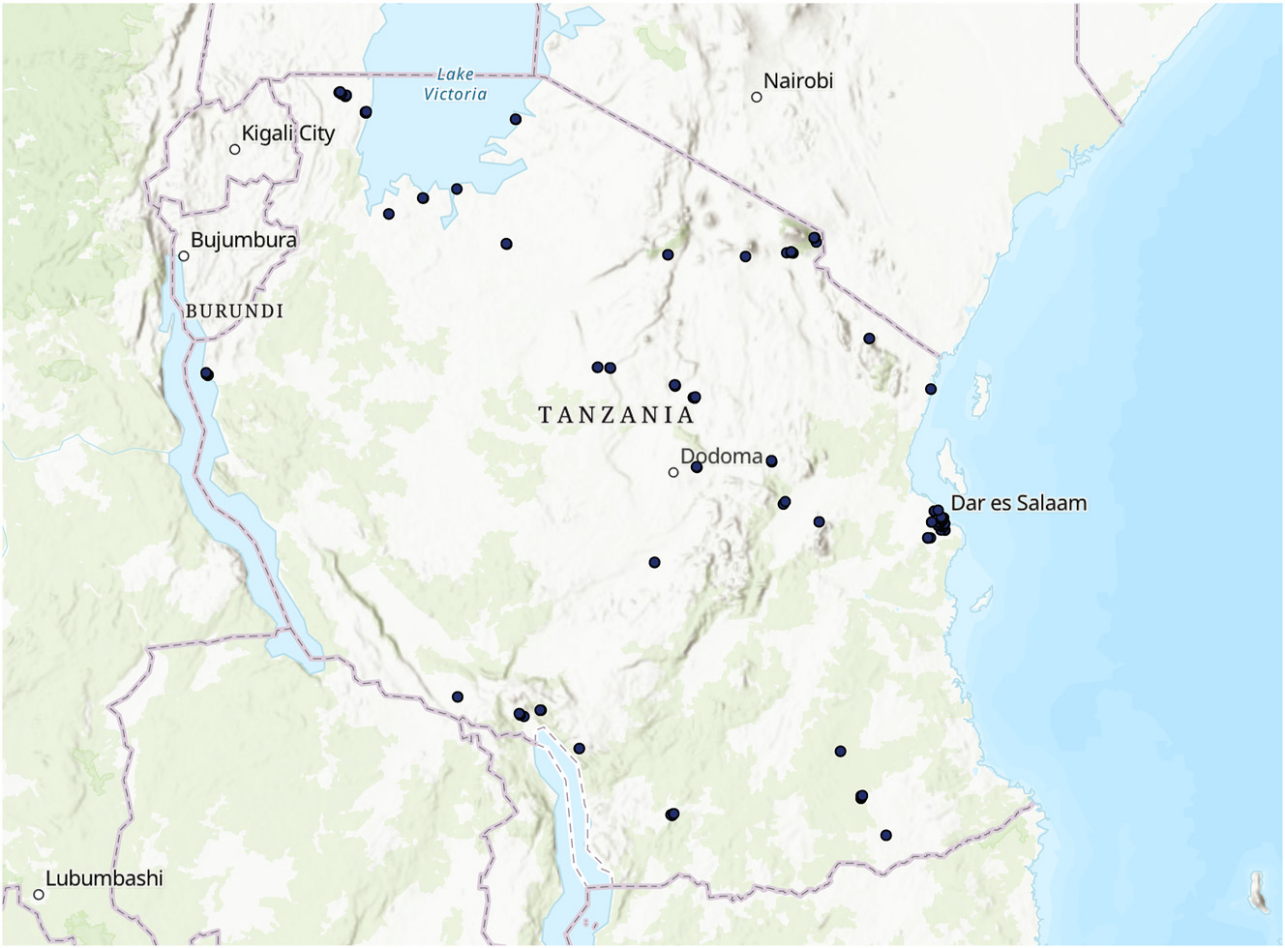
Map of Tanzania with surveyed individuals. Notes: Geographic distribution of the surveyed individuals in Tanzania.

In this paper, we use representative survey data of approximately 1200 individuals in Tanzania, which was collected in 2017 by Research ICT Africa. The survey was conducted using electronic Android tablets and an external GPS device to capture the exact coordinates of the household.5For details on the representativeness, sampling, and data collection see https://www.datafirst.uct.ac.za/dataportal/index.php/catalog/765.
Figure 1 shows the geographical distribution of survey respondents in Tanzania. We use the geographic coordinates to merge the survey with the other data sets including information on the availability and proximity of infrastructure. In particular, we calculate the distance between individuals in our survey and mobile money agents attached to different networks. Geo-location from mobile money agents is provided by *finmark*. We focus specifically on Tanzania because there is geo-location information for mobile money agents, which is unavailable for other Sub-Saharan African countries surveyed by Research ICT Africa, except Kenya. However, mobile money services in Kenya are highly dominated by Safaricom, which does not allow for a study of the impact of interoperability on market concentration.

**Figure 2:**
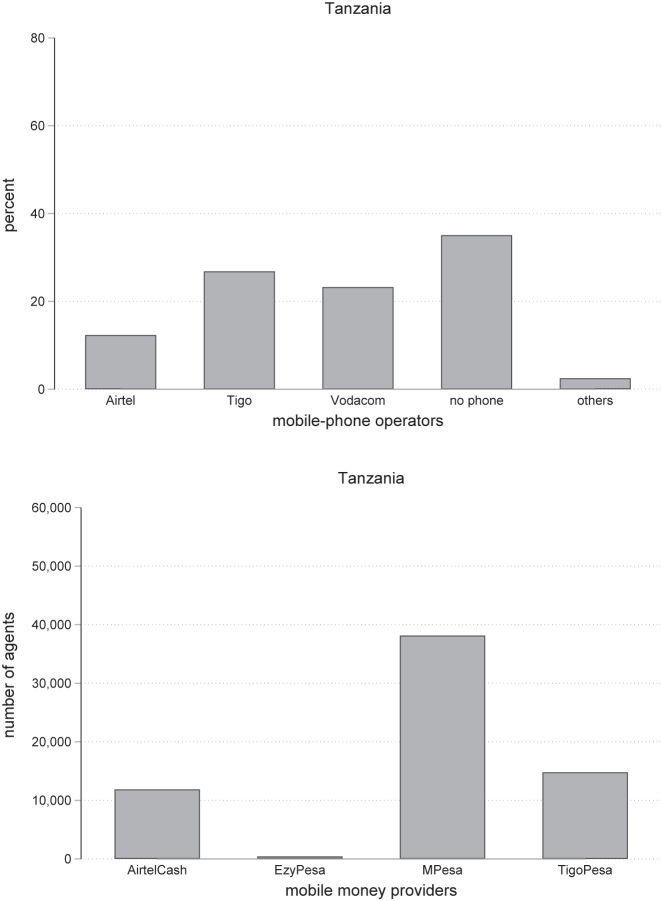
Chosen mobile-phone operator. Notes: Left: the distribution of selected mobile operators in the survey. Right: the number of mobile money agents by network operators.

Based on our survey, 34 % of individuals in Tanzania did not possess a mobile phone in 2017 (see the left panel in Figure 2). The dominant mobile money provider is M-Pesa which is served by about half of all mobile money agents in the country, as shown on the right panel in Figure 2. The shares of mobile money agents serving Airtel Cash and Tigo Pesa are 12 % and 16 %, respectfully. There are a small number of agents that serve the smallest service provider Ezy Pesa. The majority 70 % of agents serve only one provider, while about 20 % serve two different providers, and about 10 % serve three different providers.

**Figure 3:**
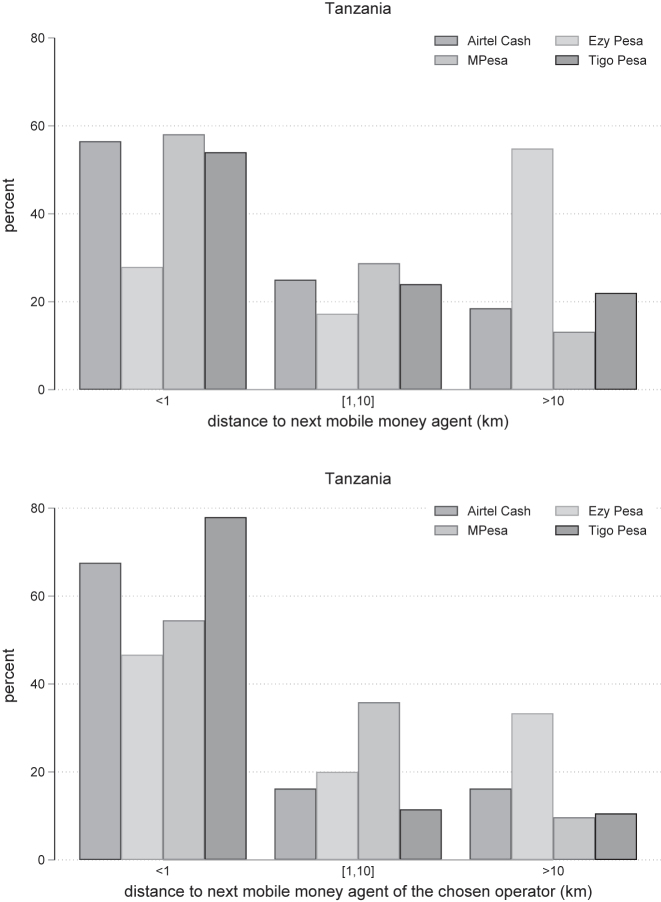
Distance to the closest mobile money agent from each provider. Notes: The distribution of distance to the closest mobile money agent from each provider.

Figure 3 shows the distribution of distances to the closest mobile money agent for each operator. The left panel shows an average distance to the mobile money agent from each operator in the whole sample of respondents. When only the sub-samples of subscribers to each operator are considered in the right figure, the average distance decreases. This suggests that distance to the agent matters when choosing an operator. Ezy Pesa has a very low market share and consequently, mobile money agents of this operator are located further away. The distributions of distances for the other operators in Tanzania are comparable. For about 60 percent of users, there is an agent of any of the providers located within 1 km. When we consider the agents of the chosen provider, this number increases to almost 80 percent. Mobile money users do not opt for operators whose agents are far away.

Next, we use nighttime light (NTL) data as a proxy for economic development. This data stems from the Visible Infrared Imaging Radiometer Suite (VIIRS) from the *Suomi* satellite provided by the Earth Observations Group (EOG), Payne Institute for Public Policy. We apply the yearly cloud-free average data for 2016. We use nighttime light intensity averages at 15 arc-second geographic grids ( ≈465m×465m at the equator, or ≈465m×385m at 35 degrees of latitude). Outliers, such as light from the aurora, fires, boats, and other temporal lights were filtered out by EOG.

The next database comes from *OpenStreetMap* (*OSM*), a collaborative effort to set up a free database with geographic information on infrastructure. This database provides among others information about the geo-location of banking facilities.6We downloaded the data from Geofabrik’s free download server in December 2019. The final database on the cell tower location was downloaded from *OpenCelliD*.7
https://www.opencellid.org/downloads.php. In addition to tower location, the date of construction and tower technology can be observed including GSM (2G), UMTS (3G), and LTE (4G). We use only information about antennas constructed before 2017 to ensure that individuals in our survey could use these antennas. For each household, we calculate the distance to the closest antenna of each technology.

Table 1 shows summary statistics for the penetration of mobile phones, usage of banking services, NTL data, and distance to infrastructure in Tanzania for 1200 individuals in our survey. A mobile phone was owned by 66 % of individuals in the sample, whereas 20 % owned a smartphone. Moreover, 55 % used mobile money, 17 % had a bank account and 10 % had a credit card. Using mobile money, owning a bank account, and owning a credit card are not exclusive. In Tanzania, mobile coverage of the most basic technology, GSM (2G), was available for almost all individuals (96 %). In comparison, UMTS (3G) was available for almost two-thirds (64 %) and the share of individuals with LTE (4G) coverage was only 41 %. Based on the NTL satellite data, slightly more than half of the individuals in our sample lived in places that were not light at night. The light intensity was on average 4.32 with a maximum of 28.

**Table 1:** Summary statistics: Tanzania.

Variables	(1)	(2)	(3)	(4)	(5)	(6)
	mean	sd	min	p50	max	*N*
Mobile money (0/1)	0.55	0.50	0.00	1.00	1.00	1200
Bank account (0/1)	0.17	0.38	0.00	0.00	1.00	1173
Credit card (0/1)	0.10	0.30	0.00	0.00	1.00	1173
Mobile phone (0/1)	0.66	0.47	0.00	1.00	1.00	1173
Smartphone (0/1)	0.20	0.40	0.00	0.00	1.00	1173
Light intensity	4.32	6.36	0.00	0.00	28.00	1173
Distance to next bank	20.03	29.07	0.03	5.10	140.56	1173
Distance to next ATM	24.76	28.41	0.03	14.19	110.58	1173
Distance to the road network	0.70	1.21	0.00	0.28	7.66	1173
Distance to the electricity grid	15.65	30.09	0.01	3.63	167.13	1173
Distance to next railway station	62.65	87.22	0.61	15.22	371.65	1173
Distance to next bus stop	14.82	28.19	0.03	3.46	139.37	1173
Distance to next town	23.32	18.84	0.01	20.97	111.05	1173
Distance to next city	57.92	65.48	0.63	33.64	253.34	1173
LTE (4G) coverage (0/1)	0.41	0.49	0.00	0.00	1.00	1194
UMTS (3G) coverage (0/1)	0.64	0.48	0.00	1.00	1.00	1195
GSM (2G) coverage (0/1)	0.96	0.21	0.00	1.00	1.00	1200

Notes: The table reports summary statistics.

## 5 The Model

### 5.1 The Choice of Network Operator

We estimate a number of models for the decision to adopt mobile services from different operators and for usage of mobile money services. First, we estimate a multinomial logit model in which consumers choose between different network operators in Tanzania. An individual consumer *i* = 1, …, *N* chooses network operator *j* ∈ *J* when *U*_*ij*_ = max_*k*∈*J*_ *U*_*ik*_, where we define a standard linear utility which consumer *i* derives from adopting a mobile phone denoted as:

(1) $$U_{ij}=Z_{i}\beta_{j}+\xi_{j}+\epsilon_{ij}$$

Here *Z*_*i*_ includes a set of individual/household characteristics and infrastructure variables. The alternative-specific coefficients, *β*_*j*_, are estimated relative to the outside option of not having a mobile phone. The individual-specific valuation for alternative *j*, i.e. the ‘logit error term’, is represented by *ϵ*_*ij*_. It is assumed to be identically and independently distributed over network operators and individuals according to the type I extreme value distribution. Finally, *ξ*_*j*_ denotes the quality of network operator *j*, which is estimated as a network-specific dummy variable. We do not use the prices of mobile services in the estimation because we do not know the exact tariff plan used by individuals. Thus, we cannot estimate price elasticities of demand for mobile services, but *ξ*_*j*_ should control for the differences in average prices of mobile networks. In this regression, we are particularly interested in estimating how distance to mobile money agents impacts network adoption decision.

The assumption on the distribution of the error term *ϵ*_*ij*_ allows us to formulate standard multinomial logit choice probabilities. The parameters of the model are then estimated using the maximum likelihood estimator. We use the estimates to conduct simulations of how enabling agents to serve multiple mobile money providers impacts the market shares of network operators.

### 5.2 The Usage of Mobile Money

Separately from the choice of a mobile network operator, we estimate the adoption of mobile money services. The decision to use mobile money services is made in two stages. In the first stage, consumers choose one from available mobile networks or no mobile access. In the second stage, those who subscribed to mobile services decide whether to use mobile money. In the first stage, we focus on the zero-one adoption decision and ignore the decision to subscribe to different network operators. This is because the decision to use mobile money should not be impacted by the network operator to which individuals subscribe. However, the number of observations in the regressions for separate network operators is also small. Thus, the decision problem of consumer *i* can be written using the following two equations:

(2) $$Y_{i}=\left\{\begin{array}{cc} 1\; & \;\text{ if }Y_{i}^{*}=Z_{i}\beta +\epsilon_{i}>0\\ 0\; & \;\text{ if }Y_{i}^{*}=Z_{i}\beta +\epsilon_{i}\leq 0 \end{array}\right.$$

(3) $$V_{i}=\left\{\begin{array}{cc} 1\; & \;\text{ if }V_{i}^{*}=X_{i}\gamma +\varepsilon_{i}>0\\ 0\; & \;\text{ if }V_{i}^{*}=X_{i}\gamma +\varepsilon_{i}\leq 0 \end{array}\right.$$

where the first Equation (2) is similar to the network choice model described above, except that there are only two alternatives. We use the same set of individual/household characteristics and infrastructure variables, *Z*_*i*_. A mobile network subscription, *Y*_*i*_ = 1, is chosen when the utility is greater than zero, $Y_{i}^{*}>0$ . The second Equation (3) denotes the use of mobile money, which is observed only if *Y*_*i*_ = 1. The use of mobile money is determined by individual characteristics and infrastructure variables included in *X*_*i*_ with coefficient *γ*. The error term is denoted by *ɛ*_*i*_ and satisfies the condition *E*(*ɛ*_*i*_|*Z*_*i*_, *X*_*i*_) = 0. The model is non-parametrically identified from the exclusion of some of the variables in the choice equation, *Z*_*i*_, from the variables in the usage equation, *X*_*i*_. In particular, we consider that the adoption of mobile phones is determined by network coverage, which does not affect the usage of mobile money services.

There is a problem with estimating mobile money usage Equation (3) when there are unobserved characteristics of the individuals that affect both the network subscription choice and mobile money usage. Then the error term *ɛ*_*i*_ is not independent of *ϵ*_*i*_ and for a binary usage variable *V*_*i*_ a simple logit or probit estimation is not consistent. Thus, the appropriate likelihood function for the model in Equations (2) and (3) and *v*_*i*_ = 0, 1 is:

(4) $$L\left(\theta \right)=\overset{n}{\underset{i=1}{\prod }}{\left[\mathrm{Pr}\left(Y_{i}^{*}\leq 0\right)\right]}^{1-Y_{i}}\cdot {\left[\mathrm{Pr}\left(V_{i}=v_{i}|Y_{i}^{*}>0\right)\cdot \mathrm{Pr}\left(Y_{i}^{*}>0\right)\right]}^{Y_{i}}$$

Defining univariate c.d.f by Φ(*Z*_*i*_ *β*) and Φ(*X*_*i*_ *γ*) and bivariate c.d.f by $\Phi_{2}\left(Z_{i}\beta ,X_{i}\gamma ,\rho \right)=\mathrm{Pr}\left(V_{i}=1|Y_{i}^{*}>0\right)$ and $\Phi_{2}\left(Z_{i}\beta ,-X_{i}\gamma ,\rho \right)=\mathrm{Pr}\left(V_{i}=0|Y_{i}^{*}>0\right)$ , we can write Equation (4) as follows:

(5) $$L\left(\theta \right)=\overset{n}{\underset{i=1}{\prod }}{\left[\Phi \left(-Z_{i}\beta \right)\right]}^{1-Y_{i}}\cdot {\left[{\left[\Phi_{2}\left(Z_{i}\beta ,X_{i}\gamma ,\rho \right)\right]}^{V_{i}}\cdot {\left[\Phi_{2}\left(Z_{i}\beta ,-X_{i}\gamma ,\rho \right)\right]}^{1-V_{i}}\right]}^{Y_{i}}$$

where the parameter *ρ* = cov(*ϵ*, *ɛ*) denotes the covariance of the error terms from both equations and *θ* = (*β*, *γ*, *ρ*) is the vector of parameters to be estimated. We use maximum likelihood to estimate the vector of parameters *θ*.

## 6 The Estimation Results

First, we utilize a multinomial logit model, presented in the first five columns of Table 2, to estimate the impact of distance to mobile money agents on consumers’ choice of network operators (mobile money providers). Here, we assume that subscribing to a mobile operator implies the use of mobile money services from this operator. The results indicate that distance plays a significant and negative role, implying that mobile networks with fewer agents located farther away are less likely to be chosen by consumers. Notably, there are also substantial variations in the perceived utility of different networks, reflected by significant dummy variables for network operators. These differences could be attributed to variations in network quality, pricing, and coverage.

In our analysis, we have also introduced interaction terms between network dummy variables and other factors, such as network coverage by LTE technology, nighttime light data, possession of a bank account, credit card, and access to electricity. Significant differences emerge among network operators concerning these variables. Interestingly, Tigo Pesa (Tigo) is less likely to be chosen in areas where consumers are within 5 km of LTE towers, potentially due to substitution with mobile banking that typically requires mobile broadband. Other operators also exhibit a negative coefficient for LTE but are statistically insignificant. Furthermore, Tigo Pesa (Tigo) and M-Pesa (Vodacom) are less likely to be chosen in economically less developed areas, as indicated by darker nighttime light.

Consumers with a bank account are generally more inclined to subscribe to mobile services, with some variations across networks. Access to electricity also positively influences subscription rates. Payment card users are more likely to subscribe to Airtel, but no such impact is observed for other networks. We have further interacted various consumer and household characteristics with a dummy variable representing subscription to any network, as shown in Column (1). The adoption of mobile phones is influenced by factors such as consumers’ age, income level, marital status, household size, education level, and professional occupation. However, living in proximity to ATMs within 2 km does not significantly impact mobile phone adoption.

Next, we employ a two-stage Heckman selection model, presented in Columns (7) and (8) of Table 2. In the first stage, individuals decide whether to subscribe to any mobile operator (Column 7), and in the second stage, they decide whether to use mobile money (Column 8), assessed through a separate survey question. In this case, unlike in the multinomial logit, the decision to use a mobile phone does not automatically imply using mobile money. Interestingly, distance to mobile money agents is found to be insignificant in both equations. Hence, while distance matters for the choice of mobile operator, it does not significantly impact mobile phone usage.

Furthermore, the impact of proximity to LTE towers remains negative, yet statistically insignificant. Additionally, less adoption of mobile phones is observed in economically less developed areas, as indicated by darker nighttime light. Having a bank account and access to electricity positively influence mobile phone usage. Similar to the previous model, adoption of mobile phones is influenced by consumer characteristics such as age, income, marital status, household size, education, and professional activity. However, once again, living within 2 km of ATMs does not significantly impact mobile phone usage.

In the second stage, we investigate the determinants of mobile money usage among individuals who own mobile phones. Here, distance to mobile money agents significantly impacts mobile money usage after individuals acquire a mobile phone. Additionally, lower usage of mobile money is observed in darker areas. Usage tends to be higher among younger individuals, suggesting that they are more inclined to adopt mobile money services. Interestingly, no significant differences are found concerning income levels, indicating that all income groups rely on mobile money transfers. However, higher education levels are associated with increased mobile money usage. Once again, living within 2 km of ATMs does not significantly impact mobile money usage.

### 6.1 Counterfactual Simulation

We utilize our multinomial logit model from Table 2 to conduct counterfactual simulations, introducing the assumption that agents can serve multiple mobile money providers. This adjustment allows us to calculate the minimum distance to an agent from any of the network operators. In Table 3, we present the changes in market shares of mobile operators resulting from this simulation. Ezy Pesa, the smallest network operator, experiences a notable increase in its market share, rising from 1.7 % to 2.4 % (a 44.4 % increase). However, the impact on overall market concentration remains marginal. M-Pesa’s market share slightly declines from 23.9 % to 23.5 %. Additionally, we observe a marginal increase in the uptake of mobile phones.

To gain further insights, we conduct simulations considering groups of population residing in urban and rural areas, as defined during the survey.8The national census sample frames was split into urban and rural Enumerator areas (EAs). We also differentiate individuals living in areas with varying nighttime light intensity, used as a proxy for economic development. Table 3 demonstrates significant differences in market shares of operators between these different types of areas. Notably, the non-exclusivity of mobile money agents has a substantially more positive impact on the market share of Ezy Pesa in rural areas (increasing by 156.8 %) compared to urban areas (increasing by 15.3 %). Similarly, in areas identified as ’dark’ the market share of Ezy Pesa increases by 118.9 %, whereas in ‘light’ areas, it rises by 12.5 %. These variations indicate that the non-exclusivity of mobile money agents mainly benefits individuals living in rural and less economically developed areas.

Beyond its impact on market structure and competition, interoperability at the agent level also leads to a direct increase in welfare by reducing the distances traveled by consumers to access mobile money agents. Our data indicates that, on average, the distance to mobile money agents decreased from 2.92 to 2.28 km due to interoperability. Again, this reduction in average distance comes mainly from rural and less economically developed areas.

In conclusion, interoperability proves beneficial for smaller operators like Ezy Pesa, enabling them to compete to some extent. However, it does not lead to a substantial increase in their market share across the entire market or in rural and less economically developed areas. Larger networks may maintain their positions due to state dependence and factors such as network effects and switching costs, which our estimation and counterfactual simulations based on survey data in a single period cannot account for.

Our simulations reveal that market shares of large operators experience only marginal changes. Specifically, M-Pesa’s share decreases by 0.4 %, Tigo Pesa gains a mere 0.1 % share, and Airtel Cash’s share remains unchanged. Although interoperability at the agent-level does not significantly impact market structure, it holds the potential to reduce costs, as network operators no longer need to establish independent networks of agents. This could consequently result in reduced prices for consumers. However, the decision for large network operators to resist agent-level interoperability, as discussed in the introduction, ultimately hinges on how it affects their margins and profits. Our analysis does not allow us to delve into this aspect and is solely focused on assessing the potential impact of agent-level interoperability on market structure.

Moreover, our simulations suggest that market shares of large operators change only marginally. M-Pesa losses 0.4 percent share, Tigo Pesa gains 0.1 percent share and Airtel Cash’s share remains unchanged. While interoperability at the agent-level does not impact market structure, it should enable reduction in costs since network operators do not need to develop independent networks of agents. This may potentially lower the costs of providing mobile money services and reduce prices paid by consumers. However, the rationale for large network operators to oppose interoperability on the agent-level, which we discussed in the introduction, depends on how interoperability impacts their margins and profits. We are unfortunately not able to comment on this in our analysis. This study is merely focused on the potential impact of interoperability at the agent-level on market structure.

Furthermore, as discussed in Section 3, network operators in Tanzania have signed bilateral agreements to enable interoperability at the account-to-account level, theoretically reducing the significance of network effects and switching costs in market dynamics. However, questions remain about the implementation and consumer perception of interoperability.

## 7 Conclusions

In this paper, we explore the impact of interoperability between networks of mobile money agents on the choices of mobile network operators using survey data from 1200 individuals across different regions in Tanzania. The data, collected in 2017 by Research ICT Africa, is combined with information on the proximity of mobile money agents and mobile network infrastructure obtained from Open Street Map (OSM). By computing the distance from household locations to mobile money agents and network towers, we examine how this distance influences consumers’ decisions to subscribe to a specific mobile network operator (and mobile money provider). Additionally, we consider nighttime light intensity data to account for geographic variations in economic development and include a set of individual characteristics as control variables.

Our discrete choice models reveal that the distance to mobile money agents significantly affects consumers’ subscription decisions. To understand the implications of interoperability, we conduct counterfactual simulations by assuming that consumers can use the closest agent from any mobile money provider. The results indicate that interoperability at the agent level has a relatively modest impact on the market shares of mobile network operators. Smaller operators marginally gain, as their consumers can now access agents of larger providers that are often closer. We also conduct simulations considering groups of population residing in urban and rural areas, and in areas with a different level of economic development. We conclude that the non-exclusivity of mobile money agents mainly benefits individuals living in rural and less economically developed areas.

We caution that our estimation does not account for interoperability at other levels, which might be more significant and complementary to agent-level interoperability. Additionally, our findings are specific to Tanzania, where interoperability between mobile money services and the non-exclusivity of agents have been in place since 2016. Thus, the importance of distance to mobile money agents may be less pronounced in markets with established interoperability.

To the best of our knowledge, this study is the first to empirically address the role of interoperability between mobile money providers. Specifically, we analyze the effects of compatibility, particularly non-exclusivity agreements between agents and mobile money providers, on the choices and market shares of mobile money providers in the context of Tanzania. In doing so, we investigate whether dominant mobile operators may leverage downstream agent-level interoperability in the delivery of mobile money services to reinforce their upstream position in the provision of mobile services overall.

## Appendix A

**Table 2:** Multinomial logit/probit/Heckman selection model.

	Multinomial logit					Probit	Heckman	
	(1)	(2)	(3)	(4)	(5)	(6)	(7)	(8)
	Phone	Airtel	Halotel	Tigo	Vodacom		Phone	Mobile money
dist_agent	−0.012**					0.001	0.001	0.003
	(0.005)					(0.005)	(0.003)	(0.005)
Network dummies		−2.808***	−4.631***	−4.631***	−1.922***			
		(0.654)	(1.191)	(1.191)	(0.606)			
LTE <5 km		−0.137	−0.009	−1.297***	0.141	−0.332	−0.181	
		(0.446)	(1.045)	(0.384)	(0.384)	(0.349)	(0.208)	
Dark		−0.458	−0.262	−0.827**	−1.004***	−0.915***	−0.519***	−0.532**
		(0.429)	(1.078)	(0.350)	(0.369)	(0.319)	(0.192)	(0.209)
Bank account		1.279**	2.528***	2.040***	1.494***	1.694***	0.900***	0.264
		(0.613)	(0.799)	(0.554)	(0.554)	(0.533)	(0.268)	(0.290)
Credit card		2.022*	0.978	1.652	1.629	1.721	0.813	0.360
		(1.145)	(1.334)	(1.116)	(1.115)	(1.102)	(0.522)	(0.383)
Electricity		0.511**	0.284	0.861***	0.576***	0.638***	0.376***	−0.052
		(0.243)	(0.575)	(0.211)	(0.209)	(0.175)	(0.103)	(0.179)
Age <25	0.072					0.043	0.033	0.644*
	(0.356)					(0.356)	(0.211)	(0.340)
Age <35	0.703**					0.700**	0.418**	0.553
	(0.354)					(0.354)	(0.212)	(0.364)
Age <45	0.805**					0.826**	0.503**	0.612
	(0.370)					(0.370)	(0.221)	(0.381)
Age <55	0.290					0.292	0.187	0.660*
	(0.391)					(0.391)	(0.236)	(0.375)
Age <65	0.646*					0.659*	0.416*	0.630
	(0.390)					(0.391)	(0.235)	(0.405)
Income <25k	0.682*					0.700*	0.372	−0.476
	(0.406)					(0.408)	(0.246)	(0.429)
Income <100k	1.558***					1.574***	0.865***	−0.236
	(0.435)					(0.437)	(0.259)	(0.463)
Female	0.699					0.705	0.443	0.433
	(0.696)					(0.698)	(0.387)	(0.434)
Married	0.535***					0.567***	0.338***	−0.069
	(0.188)					(0.189)	(0.110)	(0.182)
HH size = 2	−0.345					−0.337	−0.217	−0.199
	(0.341)					(0.340)	(0.198)	(0.280)
HH size >2	−0.511*					−0.496*	−0.301*	−0.214
	(0.286)					(0.285)	(0.164)	(0.244)
Primary	0.487*					0.515*	0.322**	0.416
	(0.267)					(0.266)	(0.159)	(0.291)
Secondary	1.044***					1.091***	0.669***	0.730*
	(0.308)					(0.307)	(0.183)	(0.377)
Tertiary	1.196***					1.266***	0.795***	0.819*
	(0.414)					(0.413)	(0.240)	(0.430)
Employed	0.619*					0.613*	0.372*	0.323
	(0.333)					(0.333)	(0.193)	(0.261)
Self_employed	0.482*					0.407	0.247	0.316
	(0.275)					(0.275)	(0.163)	(0.235)
Housework	−0.506*					−0.555*	−0.323*	0.328
	(0.287)					(0.288)	(0.172)	(0.264)
Student	−1.001***					−1.000***	−0.580***	−0.622*
	(0.357)					(0.358)	(0.210)	(0.356)
Retired	−0.854*					−0.888*	−0.494*	0.397
	(0.454)					(0.455)	(0.268)	(0.442)
ATM <2 km	0.392					0.403	0.220	−0.094
	(0.274)					(0.275)	(0.157)	(0.187)
Constant						−1.116*	−0.660*	0.583
						(0.587)	(0.351)	(0.922)
Athrho								−0.173
Observations	5950					1190	1190	779

*denotes significance at a 10 percent level; **denotes significance at 5 percent level; and ***denotes significance at 1 percent level.

**Table 3:** Simulated market shares.

	Whole market		
	Current	Simulated	Change
Airtel Cash	12.7 %	12.7 %	−0.2 %
Ezy Pesa	1.7 %	2.4 %	44.4 %
M-Pesa	23.9 %	23.5 %	−1.5 %
Tigo-Pesa	27.2 %	27.3 %	0.2 %
None	34.5 %	34.1 %	−1.2 %

	Urban			Rural		
	Current	Simulated	Change	Current	Simulated	Change
Airtel Cash	13.8 %	13.9 %	0.2 %	11.1 %	11.0 %	−0.8 %
Ezy Pesa	2.3 %	2.7 %	15.3 %	0.8 %	2.1 %	156.8 %
M-Pesa	23.9 %	23.7 %	−0.7 %	23.8 %	23.2 %	−2.5 %
Tigo-Pesa	36.7 %	36.6 %	−0.2 %	14.2 %	14.4 %	1.5 %
None	23.3 %	23.1 %	−0.5 %	50.1 %	49.2 %	−1.7 %

	Light			Dark		
	Current	Simulated	Change	Current	Simulated	Change
Airtel Cash	14.1 %	14.0 %	−0.3 %	11.4 %	11.4 %	−0.1 %
Ezy Pesa	2.4 %	2.7 %	12.5 %	1.0 %	2.2 %	118.9 %
M-Pesa	23.3 %	23.2 %	−0.6 %	24.4 %	23.8 %	−2.3 %
Tigo-Pesa	40.5 %	40.4 %	−0.2 %	14.5 %	14.7 %	1.1 %
None	19.7 %	19.7 %	−0.3 %	48.8 %	48.0 %	−1.6 %
